# Pulses Twice a Week in Replacement of Meat Modestly Increases Diet Sustainability

**DOI:** 10.3390/nu13093059

**Published:** 2021-08-31

**Authors:** Rozenn Gazan, Matthieu Maillot, Emmanuelle Reboul, Nicole Darmon

**Affiliations:** 1 MS-Nutrition, 13005 Marseille, France; matthieu.maillot@ms-nutrition.com; 2 Aix-Marseille Univ., INRAE, INSERM, C2VN, 13005 Marseille, France; Emmanuelle.Reboul@univ-amu.fr; 3 MOISA, Université de Montpellier, CIRAD, CIHEAM-IAMM, INRAE, Institut Agro, 34060 Montpellier, France; nicole.darmon@inrae.fr

**Keywords:** pulses, french food-based dietary guideline, iron and zinc bioavailability, sustainable diets, substitution

## Abstract

The French food-based dietary guidelines recommend eating pulses at least twice a week and to reduce meat consumption. This study assessed the impact on the sustainability characteristics (nutrition, cost, environment) of individual diets of meeting the pulse guideline. Dietary data of 2028 adults from the Esteban survey were completed with the nutritional content (considering bioavailability on iron, zinc and protein), price and environmental impacts of foods. When the pulse guideline (i.e., 57 g/day) was not met, two substitution scenarios raised the quantity of pulses to the recommended level, in replacement of an equivalent portion of (i) starches or (ii) meat. Only 9.6% of the participants reached the pulse guideline. Diet sustainability characteristics improved with the meat scenario (nutritional indicators improved; diet cost, greenhouse gas emissions and acidification decreased), while several indicators deteriorated with the starches scenario. Zinc available for absorption slightly decreased in both scenarios while iron available for absorption decreased in the meat scenario only. Increasing pulse consumption to two portions/week could modestly improve the sustainability of diets when pulses replace meat but not starches. Cultural acceptability of that substitution still needs to be proven, and iron and zinc status of individuals at risk of deficiency should be monitored.

## 1. Introduction

Sustainable diets are, by definition, nutritionally adequate, economically affordable and environmentally respectful [1]. An increase or decrease in the amount consumed of specific foods may help the shift toward more sustainable diets. When consumed in excess, meat is recognized as negatively impacting both environmental and human health [2]. Therefore, studies aimed at designing more sustainable diets are often based on scenarios that replace meat and/or other animal-based products with plant-based products [3,4,5,6,7]. Diets with plenty of plant-based foods, including fruits and vegetable, nuts, pulses and wholegrains provide both health and environmental benefits [8]. Among plant-based products, starchy foods and pulses are among the lowest emitters of greenhouse gases and the cheapest foods compared to others, both expressed per 100 kcal and per 100 g [9]. Pulses (i.e., dried beans, lentils, and peas, with the exclusion of crops used mainly for oil extraction such as soybeans, and those that are harvested green), are particularly promising foods to move towards sustainable diets because they are highly nutritious, economically accessible, and their production contributes to protecting soil and the environment [10].

Pulses are nutrient-rich foods that provide proteins of good nutritional quality, complex carbohydrates, and high amounts of soluble dietary fibers, vitamins (e.g., thiamin, folate), and minerals (e.g., iron, zinc, copper, manganese, phosphorus) [11,12,13]. They are recognized as protective of human health and help the prevention and management of obesity, cardiovascular disease and diabetes [12,14,15]. Pulses are also a relatively cheap source of nutrients. In the United States, beans were shown to present the highest nutritional value per dollar [16,17], the lowest cost per gram [16], and to be among the lowest-cost sources of protein [17]. Regarding the environmental dimension, due to the ability of pulses to fix atmospheric nitrogen, the integration of pulses in crop rotations reduces the synthetic nitrogen requirements, contributing to reducing greenhouse gases emissions (GHGE) and preserving a high level of soil biodiversity [18,19].

Thanks to a nutrient-dense profile, pulses are part of food-based dietary guidelines worldwide [20]. Pulses are also a key component of healthy dietary patterns such as the Mediterranean diet [21] or the Dietary Approaches to Stop Hypertension (DASH) diet [22]. The remarkable nutritional profile of pulses allows them to be categorized in many different food groups depending on the national/regional food-based dietary guidelines. Hence, they can be grouped with either (i) vegetables and other nutrient-dense and fiber-rich plant-based foods, or (ii) meat and other protein-rich alternatives, or (iii) cereals and other starchy foods. They can also be considered as a stand-along food group [20,23,24]. In France, until recently, pulses were grouped with cereals and tubers in the “starchy” food group. In 2019, the French National Nutrition and Health Program 4 (PNNS) was updated to take sustainability issues into account. As a result, pulses are now considered a separate food category, with a general recommendation to increase their consumption. More specifically, a consumption frequency of “at least twice a week” is recommended [25,26]. A parallel recommendation is to reduce meat consumption, but there is no explicit guideline stating that pulses should (partially) replace meat [27]. In France, pulses are often consumed as a side dish for meat, and many traditional dishes containing pulses also contain meat and/or processed meat (e.g., cassoulet, sausages-lentils, couscous, etc.). Therefore, replacing meat with pulses may not seem obvious to consumers [28,29]. It is thus difficult to predict whether the expected increase in pulse consumption, if any, would replace meat or other starchy foods. Therefore, both scenarios should be considered when exploring the impacts of fulfilling the new guideline on pulse consumption in terms of diet sustainability. In addition, pulses contain bioactive compounds (e.g., phytates, saponins, tannins) known to reduce the bioavailability of key micronutrients such as iron and zinc [30] and have a lower protein quality compared to animal-based products (i.e., limited amount of certain essential amino acids [13,31] and a lower protein digestibility [31,32]) so the bioavailability of these micronutrients should also be taken into account when simulating an increase in pulse consumption.

Based on a substitution approach, the aim of this study was to assess the impact of fulfilling the French guideline on pulses (i.e., at least two portions per week) on the nutritional, economic and environmental sustainability characteristics of adult diets, considering two scenarios, i.e., pulses replacing an equivalent portion of meat or an equivalent portion of starchy foods.

## 2. Materials and Methods

### 2.1. Dietary Survey and Population Sample

This study used dietary data from the Esteban survey, a cross-sectional study that was conducted in 2014–2016 on a nationally representative sample of 2835 French adults, using a multi-stage cluster sampling technique, as previously published [33]. The aim of the Esteban survey was to describe the nutrition and health status of the French population. The survey was registered at the French National Agency for Medicines and Health Products Safety (No. 2012-A00456-34) and was approved by the Advisory Committee for the Protection of Persons in Biomedical Research. Dietary intake was assessed using three non-consecutive 24-h dietary recalls (two on weekdays and one at the weekend), within a two-week period. Participants were asked to detail in a web application or by phone, as precisely as possible, all foods and beverages consumed the day before. Biological samples were collected in a health center or at home by a nurse for assessing the nutritional status of participants. Only serum ferritin was considered in this study. The dosages of the serum ferritin were done by the Grenoble University Hospital’s Institute of Biology and Pathology using an homogeneous immunoassay [33,34]. The Esteban study provides also other individual information, such as the level of income (below 840 €/month, between 840 and 1599 €/month, between 1600 and 2499 €/month, between 2500 and 4599 €/month, higher or equal than 4600 €/month), the educational level (no diploma, high school diploma, university diploma), the level of physical activity (low, moderate, high) and the diet status, indicating whether the individual followed a specific diet (no diet, diet for medical reasons, for losing weight, vegetarian or vegan diet, other reasons).

This study used information from all participants aged between 18 and 74 years old, with both dietary data and a ferritin measurement (*n* = 2245). Under-reporting individuals, identified using the Goldberg method [35] adapted by Black et al. [36], were excluded from the analysis (*n* = 217), leading to a final sample of 2028 individuals.

### 2.2. Food Composition Database

Nutrient content, environmental impact and food prices were available for 402 foods, previously identified as widely consumed among the French population [37,38,39]. Succinctly, the macro and micronutrient content of foods were from the French Information Center on Food Quality (CIQUAL) food composition database [40]. The food database was supplemented by information required to account for the bioavailability of iron, zinc and protein [38,39]. Information on phytate and amino acid contents were extracted from the WorldFood Dietary Assessment System 2 [41], heme iron was extracted from the literature [42,43] and polyphenols from beverages were expressed as black tea equivalents with the conversion factors reported in Armah et al. [44]. GHGE (in carbon dioxide equivalents, g CO_2_ eq /100 g), atmospheric acidification (in sulfur dioxide equivalents, g SO_2_ eq /100 g) and marine eutrophication (in nitrogen equivalents, g Neq/100 g) have been estimated by the environmental consulting firm Bio By Deloitte for these 402 foods [45]. Average prices of the 402 foods were estimated based on the 2006 Kantar Worldpanel database, which provides the annual food expenditures of a representative sample of 12,000 French households [46], using a previously described methodology to estimate the mean price of each food in euros per 100 g of edible portion [47].

### 2.3. Categorization and Portion Sizes of Foods Declared as Consumed in Esteban

All foods and beverages declared in the Esteban survey (*n* = 2125) were categorized into eight groups, 26 subgroups and 34 families described in Supplemental Table S1. The Starches group included three subgroups: Refined starches (including the Bread and the Pasta/rice and semolina families), Unrefined starches (including the Potatoes, the Pulses and the Wholegrain cereals families) and Breakfast cereals. Pulse food items were “flageolets”, “red beans”, “split peas”, “chickpeas”, “cooked lentils”, “white beans”, “black beans”, “fava beans” and “lupin beans”. The Meat subgroup contained six food families: Ruminant meat (beef and lamb), Pork, Poultry and game, Cooked ham, Processed meat and Offal.

The portion size for foods from the Pulses, Pasta/rice and semolina and Potatoes families was 200 g (cooked weight) [48]. A portion of 100 g was assigned for foods from the Ruminant meat, Pork, Poultry and game and Offal families [49] and a portion of 25 g was assigned to the Cooked ham and Processed meat families [50].

### 2.4. Categorization and Portion Sizes of Foods Declared as Consumed in Esteban

In the Esteban survey 2125 foods were declared, but the food composition was available for 402 foods. Using manual food matching, 525 foods from the Esteban survey were associated with the 402 foods. Each remaining food (*n* = 1598) was assigned the nutritional, environmental and price characteristics of its food family. Characteristics of the food families were estimated as the average nutritional, environmental and price data of their related foods in the 402 food list, weighted by gender-specific food consumptions.

### 2.5. Nutritional Characteristics of Diets

Daily nutrient intakes were estimated for each individual as the sum of nutrient intakes over the survey divided by the number of recalls. Bioavailability was considered for iron, zinc and proteins, accounting for the composition of each individual diet and (for iron) individual serum ferritin level. For zinc and iron, bioavailability refers to the estimation of the absorption rate, which is then multiplied by the crude intake to assess the absorbed intake. The absorption rate was estimated using previously published algorithms which depend on positive and negative modulators, as described in Table 1. For proteins, bioavailability refers to their quality, accounting for the digestibility and biological value estimated using the protein digestibility-corrected amino acid score (PDCAAS, %) [51]. The coverages of the requirements for the nine indispensable amino acids (AA) were assessed through the ratio between their intake and their requirement [52]. Equations are fully described in Supplemental Material S1.

### 2.6. Diet Quality Indicators

The simplified Programme National Nutrition Santé Guideline Score 2 (sPNNS-GS2) score, a food-based dietary index varying from -17.0 to 13.5, was estimated for each diet to assess its adherence to the most recent French food-based dietary guidelines [48]. The Mean Adequacy Ratio (MAR, in %), estimated for each diet as the mean percentage of daily recommended intakes for 23 nutrients [54], was used as an indicator of good nutritional quality. The Mean Excess Ratio (MER, in %) and the energy density from solid foods (SED) were used as indicators of low nutritional quality. MER was estimated as the mean daily percentage of maximum recommended values for sodium, saturated fatty acids and free sugars [55]. SED was calculated for each diet by dividing energy intake from all foods, except milk and beverages, by the corresponding ingested weight [55].

### 2.7. Stratification of the Population According to the Fulfilment of the Recommended Guideline for Pulses

The total amount of pulses consumed was estimated for each individual, taking into account pulses consumed per-se and pulses from mixed dishes using recipes. Individual diets reaching the recommended guideline for pulses over the three non-consecutive 24-h dietary recalls were considered adequate (referred below as Adeq diets), while the other diets were considered inadequate (referred below as InAdeq diets). Given that the standard portion of pulses was 200 g, the recommended guideline for pulses (i.e., twice a week) corresponded to 400 g per week, 171 g for three 24-h recall days (((200 × 2)/7) × 3) and 57 g per day.

### 2.8. STARCHES and MEAT Scenarios

For each observed InAdeq diet, two substituted diets were obtained, one after the replacement of starch products with pulses (STARCHES scenario) and the other after the replacement of meat products with pulses (MEAT scenario). For each InAdeq diet, the quantity of pulses was raised to the recommended guideline for pulses (i.e., at least 171 g over the three days), and an equivalent portion of starches or meats was removed in the STARCHES and MEAT scenarios, respectively. Note that the substitutions were made in an “iso-portion” way, meaning that the quantity of meat to be removed was lower than the quantity of starches removed. This is due to the fact that the portion size of meat (i.e., 25 g for Cooked ham and Processed meat and 100 g for the other meat families) was lower than the portion size of Pulses (200 g), whereas the portion size of Pasta/rice and semolina, Potatoes and Pulses were equal. In the STARCHES scenario, a priority order was applied by removing amounts from the food families Pasta/rice and semolina first, then Potatoes and lastly the amount of grains or potatoes coming from Mixed dishes. In the MEAT scenario, the following food families were removed in this priority order: Ruminant meat (beef or lamb), Processed meat, Cooked ham, Pork and Poultry and game and, finally, the amount of meat coming from Mixed dishes. The priority order was chosen to be in line with French food-based dietary guidelines, in which a reduction of meat (from beef, lamb, pork) and processed meat is recommended for human health and for environmental concerns. Where a diet did not contain enough starches or meat to be removed, the recommended guideline for pulses was not fully reached.

### 2.9. Statistical Analyses

The percentage of Adeq diets was estimated in the population. Socio demographic and behavioral characteristics, and serum ferritin status (i.e., “depletion”: serum ferritin below 15 µg/L; “low”: between 15 µg/L and 29 µg/L; “normal”: higher or equal than 30 µg/L) were compared between individuals with Adeq and InAdeq diets, using a Chi-Squared test for qualitative variables and a generalized linear model for the age. In observed diets, the mean quantity of pulses, energy intake, diet quantity, macronutrient intakes, diet quality (sPNNS-GS2, MAR, MER SED), environmental indicators (GHGE, atmospheric acidification and marine eutrophication), diet cost, amino acids (in % of amino acids requirements), zinc and iron available for absorption, PDCAAS and modulators of bioavailability of iron and zinc, were compared between Adeq and InAdeq diets using generalized linear models, adjusted on energy intake, gender and the diet status (indicating whether the individual followed a specific diet).

The mean quantities of pulses, whether or not they were from mixed dishes, and mean quantities of food families from the Refined starches, Unrefined starches and Meat subgroups were estimated in the InAdeq diets and in the two substituted diets obtained with the STARCHES and MEAT scenarios. The impacts on the nutritional (including nutrient bioavailability) and environmental characteristics of diets, as well as diet cost, of reaching the recommended guideline for pulses were estimated in both scenarios, by comparing the substituted diets to the observed ones, using generalized linear models adjusted for energy intake. All variables of interest were also compared between both scenarios using generalized linear models adjusted for energy intake. Within observed diets and the two substituted diets obtained with the STARCHES and MEAT scenarios, the prevalence of inadequate intakes of absorbed iron and absorbed zinc were estimated by assessing the proportion of individuals whose nutrient intakes was below the recommended value for that nutrient. For each individual, the recommended amount of zinc to be absorbed to compensate for the total losses was estimated with an equation relating physiological requirement to body weight [56,57]. The requirements for absorbed iron were 0.95 mg/day for men and 1.1 mg/day for women as estimated by the French Agency for Food, Environmental and Occupational Health and Safety (ANSES) based on the distribution of mandatory losses [57].

A *p*-value of 5% was used as the threshold of significance. All statistical estimations accounted for the survey design of the Esteban survey to ensure the national representativeness of the sample. Analyses were conducted using SAS version 9.4 (SAS Institute, Cary, NC, USA). Adjusted mean values are presented in results, and raw mean values are in Supplemental Tables S2–S6.

## 3. Results

### 3.1. Adeq and InAdeq Observed Diets

Only 9.6% of the adults (*n* = 175) reached the recommended guideline for pulses over the 3-day recall and were therefore considered as Adeq (Table 2). The average content of pulses in the Adeq diets was 88 g/day (Table 3). Individuals with an Adeq diet were more likely to be men (67.5% and 45.2% of men among individuals with Adeq and InAdeq diets, respectively). No significant differences were found between individuals consuming Adeq and InAdeq diets for the serum ferritin status, socio demographic and behavioral variables (i.e., physical activity and the diet status) (Table 2).

**Figure 1:**
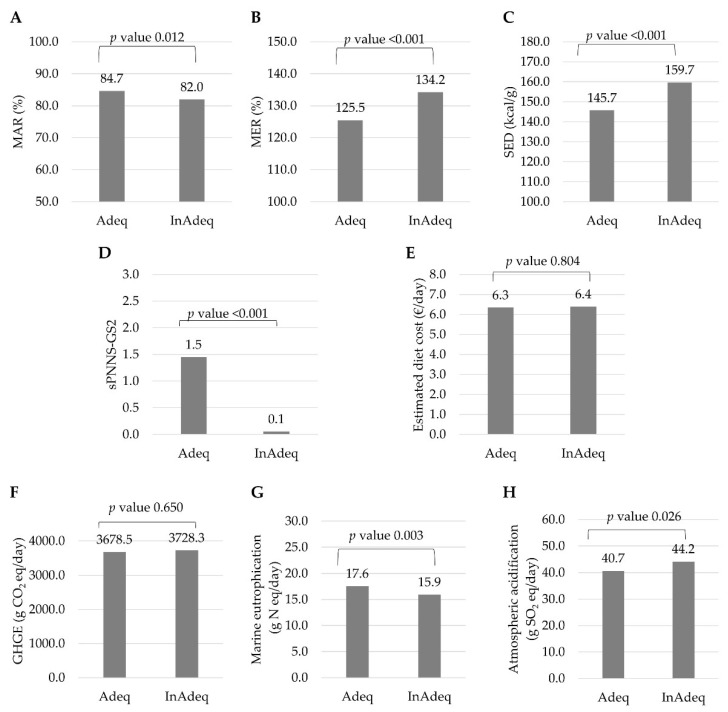
Nutritional quality (panel **A**: mean adequacy ratio; panel **B**: mean excess ratio; panel **C**: solid energy density; panel **D**: sPNNS-GS2) and environmental (panel **F**: greenhouse gas emissions; panel **G**: marine eutrophication; panel **H**: atmospheric acidification) indicators and estimated diet cost (panel **E**) of observed diets fulfilling (Adeq) and not fulfilling (InAdeq) the recommended guideline for pulses (equivalent to 57 g/day). Values are means adjusted for energy intake, gender, and diet status. MAR, Mean adequacy score; MER, Mean excess ratio; SED, Solid energy density; sPNNS-GS2, simplified Programme National Nutrition Santé Guideline Score 2; GHGE, Greenhouse gas emission.

Adeq diets provided significantly more energy (2145 kcal/day vs 1980 kcal/day respectively) and more foods than InAdeq diets, after adjustment for gender and diet status (Table 3). After adjustment for energy intake, gender, and diet status, Adeq diets had higher MAR, sPNNS-GS2 and fibers and lower MER, SED and free sugars (in % energy) than InAdeq diets, showing their better nutritional quality (Table 3 and Figure 1), except that sodium was higher in Adeq compared to InAdeq diets (Table 3). Adeq diets also had higher α-linolenic acid, magnesium, manganese, potassium, vitamin B1, B6 and B9 contents compared to InAdeq diets (Supplemental Table S4). Differences were not significant for the other micronutrients and essential fatty acids (Supplemental Table S4). Compared to InAdeq diets, Adeq ones contained more total iron, and non-heme iron, vitamin C (positive modulator), but more phytates (negative modulator). Iron available for absorption did not differ between Adeq and InAdeq diets (Table 3). For zinc, the absorption rate was lower for Adeq diets, explained by significantly more phytates, leading to lower zinc available for absorption compared to InAdeq diets (Table 3). The average PDCAAS was lower for Adeq diets compared to InAdeq diets, but all AA requirements were covered in both InAdeq and Adeq diets. Diet cost was not different between InAdeq and Adeq diets (Figure 1). Regarding the environmental impacts, Adeq and InAdeq diets had similar GHGE, but atmospheric acidification was lower and marine eutrophication higher in Adeq compared to InAdeq diets (Table 3, Figure 1).

### 3.2. STARCHES and MEAT Substitution Scenarios

In InAdeq diets, the mean content of pulses was 6 g/day (Table 4), meaning that the substitution approach aimed to add 51 g/day of pulses on average to reach the recommended level of 57 g/day, replacing foods from either the Starches food groups or the Meat subgroup, depending on the scenario. In fact, the average amount of pulses (including pulses from mixed dishes) reached with the STARCHES and MEAT scenarios was not exactly equal to 57 g/day, because some observed diets (*n* = 247 and *n* = 38 in the STARCHES and MEAT scenarios, respectively) did not contain enough starches or meat to be substituted to reach the recommended guideline for pulses. In the STARCHES scenario, the Pasta/rice and semolina family decreased from 60 g/day to 27 g/day and the Potatoes family decreased from and 53 g/day to 40 g/day (Table 4). In the MEAT scenario, the Meat subgroup decreased, from 117 g/day to 97 g/day, mainly driven by a decrease in the Ruminant meat family, in accordance with the order of priority imposed in the substitution scenario.

**Figure 2:**
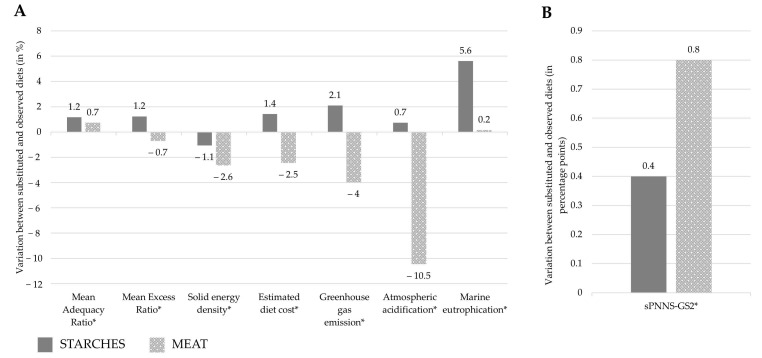
Variation in % of nutritional quality, estimated diet cost and environmental indicators (panel **A**) and in percentage points for sPNNS-GS2 (panel **B**) between diets obtained with the STARCHES scenario (iso-portion replacement of starches with pulses until the French recommended guideline for pulses is reached) or with the MEAT scenario (iso-portion replacement of meat with pulses until the French recommended guideline for pulses is reached) and the observed InAdeq diets (observed diets not reaching the recommended guideline for pulses, i.e., 57 g/day) (*n* = 1853). Values are means adjusted for energy intake. sPNNS-GS2, simplified Programme National Nutrition Santé Guideline Score 2. *: indication of a significant difference (*p* value < 0.05) between diets obtained with the STARCHES or MEAT scenarios and observed InAdeq diets, after adjusting on the energy content of diets.

Compared to the InAdeq observed diets, energy content decreased with the STARCHES scenario, while it increased with the MEAT scenario (−24 kcal/day and +28 kcal/day respectively, Table 5). After adjustment on energy content, the MEAT scenario improved all the overall nutritional quality indicators: sPNNS-GS2 and MAR increased, MER and energy density decreased (by 0.8 points, +0.7%, −2.6%, −2.5%, respectively), compared to observed InAdeq diets (Figure 2). In contrast, with the STARCHES scenario, the MER increased (i.e., nutritional quality deteriorated), in line with the increase in sodium and free sugars (shown Table 5). The three other nutritional quality indicators improved with the STARCHES scenario but the magnitude of the improvement was lower than with the MEAT scenario, except for the MAR (*p* value < 0.001).

Fibers were in line with overall indicators because they increased with both substitution scenarios (+2.3 g/day and +3.0 g/day in diets obtained with the STARCHES and MEAT scenarios, respectively). However, even if MER decreased in the MEAT scenario, due to the reduction of saturated fats, sodium increased by +76 mg/day but in a lower extent than in the STARCHES scenario (+131 mg/day) (Table 5).

Total iron and non-heme iron increased with both scenarios. However, iron available for absorption decreased in diets obtained with the MEAT scenario (−0.1 mg/day, i.e., −8.2%), because of an increase in phytates and a decrease in heme iron. When considering absorbed iron, the estimated prevalence of inadequate iron intakes was 71.4%, 70.3% and 77.1% with observed, MEAT, STARCHES diets. Less zinc was available for absorption after substitution, in particular in diets obtained with the MEAT scenario (−0.2 mg/day, being −7.7% for MEAT and −0.05 mg/day, i.e., −1.7% for STARCHES). The prevalence of inadequacy for absorbed zinc was 78.2% in observed diets and increased in both scenarios (80.7% in STARCHES scenario and 86.9% in MEAT scenario). PDCAAS decreased after both substitution scenarios, without impairing the coverage of amino acid requirements, which remained covered after substitution.

Diet cost decreased with the MEAT scenario (–2.5%) but it increased with the STARCHES one (+1.4%). The three environmental indicators deteriorated (i.e., increased) with the STARCHES scenario (+2.1%, +0.7%, +5.6% for GHGE, acidification and eutrophication, respectively). In contrast, with the MEAT scenario, GHGE and atmospheric acidification improved (i.e., decreased) and eutrophication remained almost unchanged (–4%, –10.5%, +0.2%, respectively).

## 4. Discussion

Only 9.6% of individuals spontaneously fulfilled the French food-based dietary guideline on pulses (i.e., at least twice a week, corresponding to an average of 57 g/day), and their diets had a better nutritional quality (except for sodium), similar cost and similar GHGE than observed diets not meeting the guideline. When substitution scenarios were applied to the remaining 91.4% of individuals so that their diets reached the recommended guideline for pulses, the overall sustainability of diets modestly improved (nutritional quality increased, cost and the environmental impacts decreased), but only when pulses replaced meat and processed meat. However, the zinc available for absorption was slightly reduced in both scenarios, and iron available for absorption decreased when meat was replaced with pulses, leading to an increase in the prevalence of inadequate intakes of absorbed iron and absorbed zinc.

The positive association between the consumption of pulses and the nutritional quality of diets had already been noticed in other studies. In Sweden, Canada and the U.S., consumers of pulses (or legumes in Sweden, i.e., the sum of pulses, fresh beans and soybean) compared to non-consumers had higher energy, as well as fiber, total iron, folate and magnesium intakes, adjusted for energy intake [58,59,60]. Consumers of pulses also had higher potassium and folate intakes in Sweden and Canada compared to non-consumers. Sodium intake was assessed in Canada only, and consumers of pulses had a higher sodium intake compared to non-consumers [58], in agreement with our results. However, conversely to our study in which individuals with an adequate diet for pulses were mostly men, gender was not associated with the consumption of pulses in other countries [58,59,60,61].

Substitution approaches have the advantage of isolating the impact of replacing foods or food groups with an alternative one, chosen for specific characteristic(s) (e.g., low-cost, environmentally-friendly), on the dimensions of diet sustainability [62]. As expected, thanks to a nutrient-dense profile, replacing meat or starches with pulses did not impair the nutritional quality of the diets, except an increase in sodium, and even slightly improved it. The canning process, requiring the addition of salt [11], may explain the increase in sodium after meat or starches are replaced with pulses. To our knowledge, only one substitution study focused on pulses [63]. In this previous study conducted in Sweden, replacing 50% of meat with the same amount of pulses maintained the nutritional content of diets within the Nordic nutritional recommendations, and increased the contents of fibers and folates, while reducing GHGE and land use by around 20% [63]. But, in this Swedish study, as well as in most substitution studies on diet sustainability in which animal-based products were replaced with plant-based ones [3,4,5,64,65], the assessment of the nutritional dimension did not consider the bioactive compounds contained in some plant-based products. Such bioactive compounds, including phytates, polyphenols and tannins, are present in large amounts in pulses [13]. These bioactive compounds can impair the bioavailability of some micronutrients including iron, zinc or fat-soluble vitamins [66,67]. Therefore, conclusions may be different when considering micronutrient bioavailability. In our study, an increase in pulse consumption led to a slight decrease in the amounts of iron and zinc available for absorption by the body, when bioavailability was estimated as accounting for the composition of each individual diet and individual serum ferritin level. Regarding iron, the impact of the substitution was mainly observed in the MEAT scenario, because of the decrease in heme iron and in the proportion of total meat and fish, together with an increase in phytate amounts. In a previous French study, the animal-to-plant protein ratio explained around 17% of the variation of iron absorption, but only poorly explained the variability for zinc absorption (1.6%) [39], which mainly depends on the phytate content of the diet. Our results highlight the necessity to examine nutrient bioavailability when replacing animal-based products with plant-based foods, in particular in studies with large dietary changes. However, based on Armah et al. algorithm [44], we did not discriminate the different forms of non-haem iron, in particular the presence of plant-ferritin in pulses, when estimating iron available for absorption. Plant-ferritin is currently recognized as having a better bioavailability than the other forms of non-haem iron, but more studies are needed to estimate the content of ferritin in foods, and to understand the stability and mechanisms of absorption of animal- and plant-ferritin [68,69].

In the MEAT scenario, the reduction of meat was about 15%, but the GHGE reduction was only 4%, which is far below the reduction obtained in other plant-based substitution studies. In Sweden, GHGE decreased by 20% when 50% of the quantity of meat was replaced with legumes [63]. In the Netherlands, 30% or 100% of meat and dairy amounts replaced with plant-based alternatives led to 14% and 47% GHGE reduction, respectively [3]. Whereas our aim was to simulate an increase in pulses to reach the recommended guideline for pulses, in the Swedish and Dutch studies, substitution scenarios were based on high meat reductions, which may impair acceptability. In our study, we chose to apply an iso-portion substitution to be closer to what a consumer would do, unlike the two iso-quantity substitution studies cited above. Nevertheless, our results indicate that replacing meat with pulses had a positive impact on the environmental indicators, which is not the case where pulses replaced starches.

Our results clearly show that replacing meat with pulses seems to be the most relevant change for improving diet sustainability compared to replacing starches with pulses. Yet, the central place of meat in a dish among French consumers may be a barrier to replacing meat with pulses [28]. A study conducted in France used an indirect approach to identify how French non-vegetarian consumers structure their main dish, and they specifically assessed the place of pulses [28]. In this study, individuals had to select three foods to compose a main dish, considering particular situations (e.g., an everyday meal at home, dinner with a vegetarian) [28]. Pulse-based products were more often associated with meat than with cereals or tubers, which may be a consequence of cultural habits but also a result of the previous French food-based dietary guidelines, where pulses were grouped with cereal-based products and tubers under the same group previously named “starches”. Most French consumers are unfamiliar with pulses; they find them difficult to prepare and/or consider them as vegetarian-specific food products [29]. Because our study indicates that the impact on the sustainability of diets would differ according to the way in which pulses were consumed (pulses as a replacement for meat or starches), it is important that the message on pulse consumption frequency in the French food-based dietary guidelines is complemented with practical examples of how to prepare pulse-based dishes and meals.

This study has strengths and limitations. The first strength is that the percentage of individuals reaching the recommendation for pulses was precisely estimated by quantifying pulse consumption from the multiple 24-h dietary recalls and considering pulses from mixed dishes. Another strength is that several diet sustainability dimensions were evaluated. Gathering all of the data required to assess each sustainability dimension in a unique database, as we did in this study, is not an easy task [70]. Because no existing surveys in France combine food consumption data, nutritional status (in particular individual serum ferritin status) and nutritional food composition, including information that takes bioavailability into account, as well as environmental indicators and food prices, we were limited to collecting the information missing from the Esteban survey from various sources. As a second limit, the recommended guideline for pulses was a frequency guideline (twice a week) but did not specify any recommended portion size. We used a portion size of 200 g, as proposed in Chaltiel et al. to calculate the sPNNS-GS2 score [48], but this portion size is slightly higher than the median portion size (175 g) estimated among consumers of pulses in the Esteban study (taking into account pulses in mixed dishes). Note that a lower food portion size would result in a lower impact of the substitution on the nutritional, environmental and economic dimensions. In addition, this study may appear country-specific due to the use of French data and French FBDGs. However, it is likely that the modest increase of sustainability when meat is replaced by pulses would also be observed using food consumptions from another country. A third limit was that the environmental data used were estimated using a hybrid method combining input/output and the LCA approach, as published in Bertoluci et al. (2016) [45]. A large environmental database has been very recently published in France [71], with some differences in the environmental impact of meat products compared to our environmental values for ruminant meats. A sensitivity analysis was conducted using the GHGE values of meat products from this recent database. Replacing meat with pulses to reach two portions of pulses per week using recent GHGE values for ruminants led to a 10% GHGE reduction, strengthening the positive impact of iso-portion replacement of meat with pulses on environmental indicators, as already observed in our results. As a last limit, the life cycle assessment applied for this study to derive the environmental impacts of foods did not account for crop rotations. We showed that marine eutrophication, usually due to excessive use of nitrate fertilizer, increased after iso-portion replacement of meat with pulses. However, pulses have the ability to fix air nitrogen in the soil, boosting soil fertility and reducing the need for mineral fertilizers when integrated into crop rotations [63]. Therefore, the environmental benefits of pulses may be underestimated.

## 5. Conclusions

Increasing pulse consumption to two portions per week, as a substitute for meat instead of a substitute for starches, represents a small but significant step to improving the overall nutritional quality while reducing the environmental impacts of diets, without increasing their cost. However, cultural acceptability of such a dietary change still needs to be proven, and iron and zinc status among individuals at risk of deficiency should be monitored.

## Figures and Tables

**Table 1:** Positive and negative modulators used in algorithms to assess the iron and zinc bioavailability in individual diets.

Nutrient	Algorithm to Estimate the Absorption Rate	Positive Modulators	Negative Modulators
Iron	[44]	Vitamin C (mg/day), Total meat and fish (g/day)	Serum ferritin (μg/L); Number of cups of black tea equivalents; Phytate (mg/day); Calcium (mg/day); Nonheme iron (mg/day)
Zinc	[53]	Total dietary zinc (mmol/day)	Phytates (mmol/day)

**Table 2:** Demographic, socio-economic, behavioral variables and proportion of individuals by serum ferritin status (%) among individuals with observed diets fulfilling (Adeq) and not fulfilling (InAdeq) the recommended guideline for pulses (equivalent to 57 g/day).

	All (*n* = 2028)	Adeq (*n* = 175, 9.6%)	InAdeq (*n* = 1853, 90.4%)	*p* Value *
Age (mean ± SE)	47.3 ± 0.62	49.8 ± 1.84	47.1 ± 0.58	0.208
Gender (%)				<0.0001
Men	47.4	67.5	45.2	
Women	52.6	32.5	54.8	
Income (%)				0.573
below 840 €/month	4.3	4.7	4.3	
between 840 and 1599 €/month	15.7	20.9	15.2	
between 1600 and 2499 €/month	26.7	25.6	26.8	
between 2500 and 4599 €/month	38.0	37.1	38.0	
higher or equal than 4600 €/month	15.3	11.7	15.7	
Educational level (%)				0.652
No diploma	8.8	7.1	9.0	
High school	59.3	62.6	58.9	
University	31.9	30.2	32.1	
Physical activity level (%)				0.943
Low	36.4	35.1	36.5	
Moderate	50.1	50.4	50.1	
High	13.5	14.5	13.4	
Diet status (%)				0.052
No diet	76.0	80.3	75.6	
For medical reason	3.2	2.6	3.3	
For losing weight	8.0	3.8	8.4	
Vegetarian or vegan diet	0.8	3.1	0.6	
Other reason	12.0	10.2	12.2	
Serum ferritin status (%)				
Depleted iron store	7.1	3.6	7.4	0.2868
Low iron store	9.7	9.2	9.7	
Normal iron store	83.3	87.2	82.8	

SE, Standard error of the mean. * Test of the difference of individual characteristics between Adeq and InAdeq individuals, using a linear generalized model for the age, and Chi-Squared test for qualitative variables.

**Table 3:** Nutritional characteristics of observed diets fulfilling (Adeq) and not fulfilling (InAdeq) the recommended guideline for pulses (equivalent to 57 g/day). Values are adjusted * means and standard errors.

Nutritional Characteristics	Adeq (*n* = 175)		InAdeq (*n* = 1853)		
	Adj.Mean	SE	Adj.Mean	SE	*p* Values ^†^
Pulses intake (g/day)	88.3	4.6	5.4	0.9	<0.001
Energy intake (kcal/day)	2145.1	74.1	1980.0	45.6	0.017
Diet quantity (g/day)	2988.7	93.5	2835.9	67.0	0.069
Proteins (% energy)	16.6	0.5	16.3	0.3	0.400
Carbohydrates (% energy)	46.7	0.8	45.5	0.5	0.107
Total fats (% energy)	33.2	0.6	35.6	0.3	0.001
Saturated fats (% energy)	11.8	0.3	13.7	0.2	<0.001
Free sugars (% energy)	9.1	0.7	10.9	0.5	0.004
Sodium (g/day)	3903.5	125.8	3608.4	85.9	0.003
Fibers (g/day)	26.8	0.8	19.8	0.5	<0.001
Iron (mg/day)	13.9	0.4	12.5	0.2	<0.001
Heme iron (g/day)	1.1	0.1	1.3	0.1	0.263
Non-heme iron (g/day)	12.7	0.4	11.3	0.3	<0.001
Total meat plus fish (g/day)^ ‡^	145.7	11.1	150.8	6.9	0.617
Vitamin C (g/day)^ ‡^	117.2	7.2	100.3	4.0	0.014
Phytates (mg/day) ^¶^	1397.6	46.8	1066.4	33.9	<0.001
Polyphenols from beverages (eq. cups of tea/day) ^§^	1.6	0.1	1.7	0.1	0.602
Calcium (mg/day) ^§^	936.4	48.5	929.4	33.6	0.855
Heme iron absorption rate (%)	30.1	0.6	30.5	0.3	0.518
Non-heme iron absorption rate (%)	3.7	0.3	4.1	0.1	0.135
Iron absorption rate (%)	5.7	0.3	6.7	0.2	0.008
Iron available for absorption (mg/day)	0.8	0.1	0.8	0.1	0.541
Zinc (mg/day)	10.1	0.3	10.3	0.2	0.661
Zinc absorption rate (%)	26.1	0.3	28.2	0.2	<0.001
Zinc available for absorption (mg/day)	2.5	0.1	2.7	0.0	<0.001
Histidine (% requirement)	168.4	0.9	168.3	0.6	0.593
Isoleucin (% requirement)	169.3	1.1	168.9	0.8	0.782
Leucine (% requirement)	134.3	0.8	134.7	0.7	0.421
Lysine (% requirement)	149.4	2.4	150.6	2.0	0.168
Sulfur amino acids (% requirement)	162.1	0.9	163.2	0.7	0.014
Aromatic a.a (% requirement)	198.2	0.8	197.4	0.5	0.133
Threonin (% requirement)	158.7	1.1	158.9	1.0	0.451
Tryptophan (% requirement)	203.2	0.8	203.1	0.6	0.543
Valin (% requirement)	197.0	1.5	198.0	1.0	0.286
PDCAAS (%)	93.5	0.0	94.1	0.0	<0.001

Adj. mean, adjusted mean; PDCAAS, Protein Digestibility Corrected Amino Acid Score; a.a, amino acids; SE, Standard error of the mean * means are adjusted for energy (except for energy), gender and diet status (whether the individual followed a specific diet or not) ^†^ *p*-value of the generalized linear model to test the difference between InAdeq and Adeq diets, accounting for the survey design with adjustment on total energy intake, gender and the diet status. ^‡^ Positive modulator of iron absorption; ^§^ Negative modulator of iron absorption; ^¶^ Negative modulator of iron and zinc absorption.

**Table 4:** Mean quantities of specific food subgroups and food families in observed diets not meeting the recommended guideline for pulses (InAdeq), and in substituted diets obtained with the STARCHES scenario (iso-portion replacement of starches with pulses until the French recommended guideline for pulses is reached, i.e., 57 g/day) and the MEAT scenario (iso-portion replacement of meat with pulses until the guideline for pulses is reached).

Food Subgroups and Food Families	InAdeq Observed Diets (*n* = 1853)		Substituted diets			
			STARCHES (*n* = 1853)		MEAT (*n* = 1853)	
	Mean	SE	Mean	SE	Mean	SE
Total pulses	6.1	0.4	56.2	0.2	57.9	0.2
Pulses per se	3.0	0.3	53.2	0.3	54.8	0.3
Pulses from mixed dishes	3.1	0.3	3.0	0.3	3.1	0.3
Refined starches	142.0	3.2	109.8	2.9	142.0	3.2
Bread	82.3	2.2	82.3	2.2	82.3	2.2
Pasta/rice and semolina	59.7	2.3	27.4	1.9	59.7	2.3
Unrefined starches (without pulses)	71.8	2.2	59.2	2.1	71.8	2.2
Wholegrain cereals	19.0	0.9	19.0	0.9	19.0	0.9
Potatoes	52.8	1.8	40.2	1.7	52.8	1.8
Meat	116.6	3.2	116.6	3.2	96.5	3.1
Ruminant meats	31.9	1.6	31.9	1.6	19.2	1.3
Pork	13.6	0.9	13.6	0.9	13.0	0.9
Poultry and games	32.5	1.5	32.5	1.5	30.6	1.5
Processed meat	26.0	1.2	26.0	1.2	22.0	1.1
Cooked ham	9.7	0.6	9.7	0.6	8.8	0.6
Offals	3.0	0.4	3.0	0.4	2.9	0.4

**Table 5:** Nutritional, environmental characteristics and estimated diet cost of observed diets not meeting the recommended guideline for pulses (InAdeq), and of substituted diets obtained with the STARCHES scenario (iso-portion replacement of starches with pulses until the recommended guideline for pulses is reached, i.e., 57 g/day) and with the MEAT scenario (iso-portion replacement of meat with pulses until the recommended guideline for pulses is reached). Values are means adjusted * for energy intake and standard errors.

	Observed Diets * InAdeq (*n* = 1853)		Substituted Diets STARCHES (*n* = 1853)			Observed Diets * InAdeq (*n* = 1853)		Substituted Diets MEAT (*n* = 1853)			
Nutritional, Environmental Characteristics and Diet Cost	Adj. Mean	SE	Adj. Mean	SE	*p* Value ^†^	Adj. Mean	SE	Adj. Mean	SE	*p* Value ^‡^	*p* Value ^§^
Energy (kcal/day)	2058.2	20.1	2033.8	20.1	<0.0001	2058.2	20.1	2063.2	20.0	<0.0001	<0.0001
Total quantity (g/day)	2615.9	26.8	2631.5	26.6	<0.0001	2625.1	26.7	2652.7	26.7	<0.0001	<0.0001
MAR (% adequacy)	80.8	0.3	81.8	0.3	<0.0001	81.0	0.3	81.6	0.3	<0.0001	<0.0001
MER (% excess)	135.2	0.6	136.9	0.6	<0.0001	136.0	0.6	135.1	0.6	<0.0001	<0.0001
SED (kcal/100 g)	168.0	1.3	166.2	1.3	<0.0001	168.3	1.3	163.8	1.2	<0.0001	<0.0001
sPNNS-GS2	−0.5	0.1	−0.1	0.1	<0.0001	−0.6	0.1	0.2	0.1	<0.0001	<0.0001
Proteins (% energy)	16.9	0.1	17.3	0.1	<0.0001	16.8	0.1	16.4	0.1	<0.0001	<0.0001
Carbohydrates (% energy)	44.3	0.2	43.4	0.2	<0.0001	44.3	0.2	45.5	0.2	<0.0001	<0.0001
Total fats (% energy)	36.5	0.2	36.7	0.2	<0.0001	36.5	0.2	35.4	0.2	<0.0001	<0.0001
Saturated fats (% energy)	14.6	0.1	14.6	0.1	<0.0001	14.6	0.1	14.1	0.1	<0.0001	<0.0001
Free sugars (% energy)	11.1	0.2	11.3	0.2	<0.0001	11.2	0.2	11.1	0.2	<0.0001	<0.0001
Sodium (g/day)	3530.8	23.9	3661.9	24.5	<0.0001	3551.5	24.1	3627.8	23.8	<0.0001	<0.0001
Fibers (g/day)	17.9	0.2	20.2	0.2	<0.0001	17.9	0.2	21.0	0.2	<0.0001	<0.0001
Greenhouse gas emission (g CO2eq/day)	3852.5	34.3	3933.1	34.3	<0.0001	3874.7	34.4	3720.5	32.1	<0.0001	<0.0001
Atmospheric acidification (g SO2eq/day)	48.0	0.6	48.3	0.6	<0.0001	48.3	0.6	43.2	0.5	0.0424	<0.0001
Marine eutrophication (g Neq/day)	16.7	0.1	17.6	0.1	<0.0001	16.8	0.1	16.8	0.1	<0.0001	<0.0001
Estimated diet cost (€/day)	6.3	0.1	6.4	0.1	<0.0001	6.4	0.1	6.2	0.1	<0.0001	<0.0001
Iron (mg/day)	12.0	0.1	12.9	0.1	<0.0001	12.1	0.1	12.5	0.1	<0.0001	<0.0001
Heme iron (g/day)	1.5	0.0	1.5	0.0	<0.0001	1.5	0	1.2	0.0	<0.0001	<0.0001
Non-heme iron (g/day)	10.5	0.1	11.3	0.1	<0.0001	10.6	0.1	11.3	0.1	<0.0001	<0.0001
Total meat plus fish (g/day) ^||^	168.0	2.6	169.0	2.6	<0.0001	169.0	2.6	148.8	2.5	<0.0001	<0.0001
Vitamin C(g/day) ^||^	92.8	1.7	91.9	1.7	<0.0001	93.1	1.7	92.9	1.7	<0.0001	<0.0001
Phytates (mg/day) ^**^	922.3	8.9	1023.1	9.3	<0.0001	928.0	9.0	1078.1	9.1	<0.0001	<0.0001
Polyphenols from beverages (eq. cups of tea/day) ^¶^	1.5	0.0	1.5	0.0	0.7795	1.5	0.0	1.5	0.0	0.7795	0.7678
Calcium (mg/day) ^¶^	910.0	8.0	928.0	8.1	<0.0001	915.4	8.1	929.7	8.0	<0.0001	<0.0001
Heme absorption rate (%)	30.9	0.2	30.8	0.2	<0.0001	30.9	0.2	30.9	0.2	<0.0001	<0.0001
Non-heme absorption rate (%)	4.5	0.1	4.3	0.1	<0.0001	4.5	0.1	4.3	0.1	<0.0001	<0.0001
Iron absorption rate (%)	7.6	0.1	7.3	0.1	<0.0001	7.6	0.1	6.7	0.1	<0.0001	<0.0001
Iron available for absorption (mg/day)	0.9	0.0	0.9	0.0	<0.0001	0.9	0.0	0.8	0.0	<0.0001	<0.0001
Zinc (mg/day)	10.3	0.1	10.5	0.1	<0.0001	10.4	0.1	9.9	0.1	<0.0001	<0.0001
Zinc absorption rate (%)	29.1	0.1	28.1	0.1	<0.0001	29.0	0.1	28.1	0.1	<0.0001	0.0051
Zinc available for absorption (mg/day)	2.8	0.0	2.8	0.0	<0.0001	2.9	0.0	2.6	0.0	<0.0001	<0.0001
Histidine (%requirement)	170.5	0.3	171.9	0.3	<0.0001	170.5	0.3	169.3	0.3	<0.0001	<0.0001
Isoleucin (% requirement)	170.6	0.2	171.7	0.2	<0.0001	170.6	0.2	171.1	0.2	<0.0001	<0.0001
Leucine (% requirement)	135.7	0.1	136.4	0.1	<0.0001	135.7	0.1	136.1	0.1	<0.0001	<0.0001
Lysin (% requirement)	154.7	0.4	157.0	0.4	<0.0001	154.7	0.4	153.2	0.4	<0.0001	<0.0001
Sulfur amino acids (% requirement)	165.3	0.2	163.7	0.2	<0.0001	165.3	0.2	163.4	0.2	<0.0001	<0.0001
Aromatic amino acids (% requirement)	197.3	0.2	198.0	0.2	<0.0001	197.3	0.2	199.1	0.2	<0.0001	<0.0001
Threonin (% requirement)	160.6	0.2	161.9	0.2	<0.0001	160.6	0.2	160.1	0.2	<0.0001	<0.0001
Tryptophan (% requirement)	201.7	0.2	201.2	0.2	<0.0001	201.7	0.2	202.3	0.2	<0.0001	<0.0001
Valin (% requirement)	198.2	0.2	198.9	0.2	<0.0001	198.2	0.2	199.3	0.2	<0.0001	<0.0001
PDCAAS (%)	94.9	0.02	94.5	0.02	<0.0001	94.9	0.02	94.5	0.02	<0.0001	0.9406

Adj. mean, adjusted mean; MAR, Mean adequacy ratio; MER, Mean excess ration; PDCAAS, Protein Digestibility Corrected Amino Acid Score; SED, Solid energy density; SE, Standard error. * Adjusted mean value in observed diets were slightly different between STARCHES and MEAT scenarios because of the energy adjustment ^†^ *p* value of the generalized linear model to test the difference between STARCHES and observed diets, accounting for the survey design and adjusted on total energy (except for energy); ^‡^ *p* value of the generalized linear model to test the difference between MEAT and observed diets, accounting for the survey design and adjusted on total energy (except for energy); ^§^ *p* value of the generalized linear model to test the difference between diets obtained with the STARCHES and MEAT scenarios, accounting for the survey design and adjusted on total energy (except for energy) ^||^ Positive modulator of iron absorption; ^¶^ Negative modulator of iron absorption; ** Negative modulator of iron and zinc absorption.

## Supplementary Materials

The following are available online at https://www.mdpi.com/article/10.3390/nu13093059/s1; Table S1: Food categorization; Table S2: Average energy intake, total quantity, nutritional indicators, environmental impact and estimated diet cost between observed diets fulfilling (Adeq) and not fulfilling (InAdeq) the recommended guideline for pulses (equivalent to 57 g/day); Table S3: Bioavailability estimates and nutrient and bioavailability modulators contents between observed diets fulfilling (Adeq) and not fulfilling (InAdeq) the recommended guideline for pulses (equivalent to 57 g/day).; Table S4: Average fatty acids, vitamins and minerals intakes among observed diets fulfilling (Adeq) and not fulfilling (InAdeq) the recommended guideline for pulses (equivalent to 57 g/day) with and without adjustment; Table S5: Average total energy content, total quantity, nutritional indicators, environmental impact and estimated diet cost in observed diets not fulfilling the recommended guideline for pulses (InAdeq) and in substituted diets obtained with the STARCHES scenario (iso-portion replacement of starches by pulses until the recommended guideline for pulses is reached, i.e., 57 g/day) and with the MEAT scenario (iso-portion replacement of meat by pulses until the recommended guideline for pulses is reached).; Table S6: Bioavailability estimates and nutrient and bioavailability modulators contents in observed diets not fulfilling the recommended guideline for pulses (InAdeq) and in substituted diets obtained with the STARCHES scenario (iso-portion replacement of starches by pulses until the recommended guideline for pulses is reached, i.e., 57 g/day) and with the MEAT scenario (iso-portion replacement of meat by pulses until the recommended guideline for pulses is reached.
