# Supplementary material for: Pulses Twice a Week in Replacement of Meat Modestly Increases Diet Sustainability
Source: Nutrients. 2021 Aug 31;13(9):3059. doi: 10.3390/nu13093059 (PMC8466503; doi:10.3390/nu13093059)
Supplement: Supplementary file 1 [file nutrients-13-03059-s001.zip › nutrients-1340748-supplementary.pdf]

Supplementary material

Table S1. Food categorization

Groups (n = 8)	Subgroups (n = 26)	Families (n = 34)
Fruits & Vegetables	Raw & cooked vegetables	Raw & cooked vegetables
	Fresh & processed fruits	Fresh & processed fruits
Starches	Refined starches	Bread
		Pasta/rice & semolina
	Unrefined starches	Pulses
		Wholegrains cereals
		Potatoes
	Breakfast cereals	Breakfast cereals
Meat/fish/eggs & substitutes	Egg	Egg
	Fish	Fish
	Meat	Ruminant meat
		Pork
		Poultry & games
		Processed meat
		Cooked ham
		Offals
	Meat substitutes	Meat substitutes
Mixed dishes & sandwiches	Mixed dishes	Mixed dishes
	Sandwich & salted pastries	Sandwich & salted pastries
Dairy products & substitutes	Milk	Milk
	Yoghurt	Yoghurt
	Cheese	Cheese
	Dairy substitutes	Dairy substitutes
Sweet products	Dairy-desserts	Dairy-desserts
	Cakes & tarts	Cakes & tarts
	Biscuits & sweets	Biscuits & sweets
Water & Beverages	Water	Water
	Hot drinks	Hot drinks
	Drinks with sweeteners	Drinks with sweeteners
	Sweet drinks	Sweet drinks
	100% Fruit juices	100% Fruit juices
Fat products	Animal fats	Animal fats
	Vegetal fats	Vegetal fats
	Sauces and spices	Sauces and spices

Supplementary material

Method S1. Method to consider bioavailability for iron, zinc and protein

Estimation of iron absorption

For each individual, Armah et al. [47] algorithm was used to estimate non-heme iron absorption (%) as follows:

$$ln(\text{non-heme iron absorption in \%}) = 6.294 - 0.709\ ln(SF) + 0.119 * ln(C) + 0.006\,ln(MFP+0.1) - 0.055\,ln(T+0.1) - 0.247\,ln(P) - 0.137\,ln(Ca) - 0.083\,ln(NH)$$

where SF is individual serum ferritin (µg/L), C is vitamin C intake (mg/d), TMF is total meat plus fish (g/d), T is tea, coffee and wine (number of cups of black tea equivalents), P is phytate intake (mg/d), Ca is calcium intake (mg/d), and NH is nonheme iron intake (mg/d). Total meat and fish quantity in the diets was calculated as the sum of meat and fish foods and meat/fish as ingredients in mixed dishes.

Heme iron absorption (%) was estimated using Hallberg et al.'s equation [74] :

$$Log\ heme\ iron\ absorption\ (\%) = 1.9897 - 0.3092 \times log\ SF$$

where SF is individual serum ferritin (µg/L).

$$Total\ iron\ absorption\ (\%) = \frac{\text{heme iron intake * \% heme iron absorption +non-heme iron intake* \% non-heme iron absorption}}{total\ iron\ intake} * 100$$

For heme iron and non-heme iron absorption estimations, a cut-off value of 60 mg/L has been applied on the serum ferritin level because no homeostatic up-regulation of iron absorption occurs at a serum ferritin level higher than 60 mg/L [75, 76].

Estimation of zinc absorption

Miller et al. [73] algorithm was used to estimate zinc absorption (%) as follows:

$$AZ = 0.5 \times \left\{0.13 + TDZ + 0.10\left(1 + \frac{TDP}{1.2}\right) - \sqrt{\left(0.13 + TDZ + 0.10\left(1 + \frac{TDP}{1.2}\right)\right)^2 - 4 \times 0.13 \times TDZ}\right\}$$

$$Total\ zinc\ absorption\ (\%) = \frac{AZ * 65.4}{total\ zinc\ intake} * 100$$

Supplementary material

where AZ is daily absorbed zinc (mmol/d), TDZ is total dietary zinc intake (mmol/d) and TDP is total dietary phytates intake (mmol/d). Molar masses of 65.4 and 660 g.mol-1 were used for zinc and phytates, respectively.

Estimation of protein quality (PDCAAS)

The adult pattern used as reference, defining the amino acid/protein ratio requirements for each of the 9 indispensable amino acids, was obtained from the French Agency for Food, Environmental and Occupational Health & Safety [55]. Protein digestibility was assigned by food source based on published values [54, 77]. PDCAAS was estimated as follows[78]:

$$PDCAAS = protein\ digestibility\ (\%) \times lowest\ amino\ acid\ ratio$$

$$amino\ acid_i\ ratio = \left(\frac{mg\ of\ amino\ acid_i\ in\ 1\ g\ of\ digested\ protein}{mg\ of\ amino\ acid_i\ \ in\ requirement\ pattern}\right)$$

Supplementary material

Table S2. Average energy intake, total quantity, nutritional indicators, environmental impact and estimated diet cost between observed diets fulfilling (Adeq) and not fulfilling (InAdeq) the recommended guideline for pulses (equivalent to 57 g/d)

	**All (n = 2028)**	**Adeq (n = 175, 9.6%)**	**InAdeq (n = 1853, 90.4%)**	
	Mean ± SE	*Mean ± SE*	*Mean ± SE*	**Pvalue[1]**
Pulse amounts (g/d)	14.1 ± 0.9	89 ± 4.8	6.1 ± 0.4	<0.001
Energy (kcal/d)	2084.4 ± 21.7	2330.6 ± 64.5	2058.2 ± 20.1	<0.001
Total quantity (g/d)	2664.8 ± 32	2971 ± 88.3	2632.2 ± 30.9	<0.001
Quantity of liquids (g/d)	1445.2 ± 24.8	1537.9 ± 75.1	1435.3 ± 24.9	0.175
Quantity of solids (g/d)	1219.6 ± 13.9	1433.1 ± 40.8	1196.9 ± 12.7	<0.001
MAR (% adequacy/d)	81.7 ± 0.4	86.6 ± 1.4	81.2 ± 0.4	<0.001
MER (% excess/d)	137.2 ± 1.3	142.2 ± 3.5	136.6 ± 1.3	0.100
SED (kcal/100g)	167.8 ± 1.2	160.7 ± 3.6	168.5 ± 1.3	0.037
sPNNS-GS2	-0.5 ± 0.1	-0.1 ± 0.4	-0.6 ± 0.1	0.186
Proteins (% energy)	16.8 ± 0.1	16.9 ± 0.4	16.8 ± 0.1	0.781
Carbohydrates (% energy)	44.4 ± 0.2	45.7 ± 0.7	44.3 ± 0.2	0.054
Total fats (% energy)	36.3 ± 0.2	34.3 ± 0.6	36.5 ± 0.2	0.001
Saturated fats (% energy)	14.4 ± 0.1	12.8 ± 0.3	14.6 ± 0.1	<0.001
Free sugars (% energy)	11 ± 0.2	9.7 ± 0.5	11.2 ± 0.2	0.007
Sodium (g/d)	3635.1 ± 42	4269.6 ± 137.9	3567.5 ± 38.6	<0.001
Fibers (g/d)	18.9 ± 0.2	26.8 ± 0.8	18 ± 0.2	<0.001
Greenhouse gas emission (g CO2eq/d)	3924.8 ± 50.5	4238.7 ± 143.4	3891.3 ± 49.9	0.015
Atmospheric acidification (g SO2eq/d)	48.7 ± 0.8	50.7 ± 2.2	48.5 ± 0.8	0.290
Marine eutrophication (g Neq/d)	17.2 ± 0.2	20.3 ± 0.9	16.9 ± 0.2	<0.001
Estimated diet cost (€/d)	6.4 ± 0.1	7 ± 0.2	6.4 ± 0.1	0.015

MAR, Mean adequacy ratio ; MER, Mean excess ratio ; SED, Solid energy density

[1] *pvalue of the generalized linear model to test the difference between InAdeq and Adeq diets, accounting for the survey design without adjustment*

Supplementary material

Table S3. Bioavailability estimates and nutrient and bioavailability modulators contents between observed diets fulfilling (Adeq) and not fulfilling (InAdeq) the recommended guideline for pulses (equivalent to 57 g/d).

**Nutrients and bioavailability modulators**	**All (n = 2028)** *Mean ± SE*	**Adeq (n = 175)** *Mean ± SE*	**InAdeq (n = 1853)** *Mean ± SE*	**Pvalues[1]**
Iron (mg/d)	12.5 ± 0.2	15 ± 0.5	12.2 ± 0.1	<0.001
Heme iron (g/d)	1.5 ± 0	1.6 ± 0.1	1.5 ± 0	0.712
Non-heme iron (g/d)	10.9 ± 0.1	13.4 ± 0.4	10.7 ± 0.1	<0.001
Positive modulators				
Total meat plus fish (g/d)	171 ± 3.3	183.4 ± 12.3	169.7 ± 3.1	0.254
Vitamin C(g/d)	95.1 ± 1.7	111.8 ± 6.7	93.4 ± 1.7	0.007
Negative modulators				
Phytate (mg/d)	977.6 ± 13	1399.7 ± 52.6	932.7 ± 10.9	<0.001
Polyphenols from beverages (eq. cups of tea/d)	1.5 ± 0	1.4 ± 0.1	1.5 ± 0	0.587
Calcium (mg/d)	928.9 ± 10.8	1019 ± 38.6	919.3 ± 10.9	0.014
Heme absorption rate (%)	30.7 ± 0.2	29.6 ± 0.6	30.8 ± 0.2	**0.048**
Non-heme absorption rate (%)	4.4 ± 0.1	3.6 ± 0.2	4.5 ± 0.1	**0.001**
Iron absorption rate (%)	7.5 ± 0.1	6.1 ± 0.3	7.6 ± 0.1	**<0.001**
Iron available for absorption (mg/d)	0.9 ± 0	0.9 ± 0.1	0.9 ± 0	0.895
Zinc (mg/d)	10.5 ± 0.1	11.6 ± 0.5	10.4 ± 0.1	0.009
Zinc absorption rate (%)	28.6 ± 0.2	24.9 ± 0.5	29 ± 0.2	<0.001
Zinc available for absorption (mg/d)	2.8 ± 0	2.7 ± 0.1	2.9 ± 0	0.045
Histidine (% requirement)	170.6 ± 0.3	171 ± 0.9	170.6 ± 0.3	0.832
Isoleucin (% requirement)	170.6 ± 0.2	170.3 ± 0.8	170.6 ± 0.2	0.863
Leucine (% requirement)	135.7 ± 0.1	135.3 ± 0.5	135.7 ± 0.1	0.351
Lysine (% requirement)	154.4 ± 0.4	152.6 ± 1.5	154.6 ± 0.4	0.251
Sulfur amino acids (% requirement)	165.2 ± 0.2	164 ± 0.5	165.3 ± 0.2	0.019
Aromatic amino acid (% requirement)	197.4 ± 0.2	198.3 ± 0.6	197.3 ± 0.2	0.157
Threonin (% requirement)	160.6 ± 0.2	160.1 ± 0.7	160.6 ± 0.2	0.526
Tryptophan (% requirement)	201.8 ± 0.2	202.2 ± 0.7	201.7 ± 0.2	0.657
Valin (% requirement)	198.1 ± 0.2	197.1 ± 1	198.2 ± 0.2	0.390
PDCAAS	94.8 ± 0	94.2 ± 0	94.9 ± 0	0.001

Adeq, diets reaching the pulse's guideline of two portion of pulses per week; InAdeq, diets not reaching the recommended guideline for pulses; PDCAAS, Protein Digestibility Corrected Amino Acid Score

[1] *pvalue of the Chi-square test for serum ferritin status and pvalue of the generalized linear model to test the difference between InAdeq and Adeq diets, accounting for the survey design without adjustment*

Supplementary material

Table S4. Average fatty acids, vitamins and minerals intakes among observed diets fulfilling (Adeq) and not fulfilling (InAdeq) the recommended guideline for pulses (equivalent to 57 g/d) with and without adjustment.

	**All (n = 2028)**	**Adeq (n = 175)**	**InAdeq (n = 1853)**		**Adeq (n = 175)**	**InAdeq (n = 1853)**	
	Mean ± SE	*Mean ± SE*	*Mean ± SE*	**Pvalue[1]**	*Adj. mean ± SE*	*Adj. mean ± SE*	**Pvalue[2]**
MUFA (g/d)	29 ± 0.4	31.7 ± 1.1	28.7 ± 0.3	0.009	27.4 ± 0.7	28.5 ± 0.3	0.115
PUFA (g/d)	12 ± 0.2	13.9 ± 0.6	11.8 ± 0.2	<0.001	13.3 ± 0.4	12.7 ± 0.3	0.185
DHA (g/d)	0.1 ± 0	0.2 ± 0	0.1 ± 0	0.386	0.2 ± 0	0.2 ± 0	0.616
EPA (g/d)	0.1 ± 0	0.1 ± 0	0.1 ± 0	0.957	0.1 ± 0	0.1 ± 0	0.751
Linoleique fatty acid (g/d)	10.4 ± 0.2	12 ± 0.5	10.2 ± 0.2	0.000	11.5 ± 0.4	11 ± 0.3	0.301
α-linolenique fatty acid (g/d)	1.2 ± 0	1.5 ± 0.1	1.1 ± 0	<0.001	296.1 ± 16.3	296 ± 12.3	0.003
Cholesterol (g/d)	327.4 ± 5.4	361.3 ± 18.1	323.8 ± 5.2	0.036	1.8 ± 0.1	1.7 ± 0	0.995
Copper (mg/d)	1.6 ± 0	1.8 ± 0.1	1.6 ± 0	0.006	2534.1 ± 93.5	2393.7 ± 67.4	0.253
Water (g/d)	2221 ± 29.4	2465 ± 81.4	2195.1 ± 28.8	0.001	140 ± 5.9	143.4 ± 3.5	0.094
Iodin (µg/d)	138.7 ± 2	149.5 ± 5.8	137.6 ± 2.1	0.047	378 ± 8.6	349.4 ± 4.1	0.559
Magnesium (mg/d)	328.7 ± 3.4	383.8 ± 12	322.8 ± 3.1	<0.001	4.6 ± 0.2	4 ± 0.2	0.002
Manganese (mg/d)	3.5 ± 0.1	4.4 ± 0.2	3.4 ± 0	<0.001	1303.4 ± 38.4	1269.1 ± 23.9	0.001
Phosphorus (mg/d)	1294.4 ± 12.8	1452.8 ± 44.4	1277.6 ± 12.1	<0.001	3353.9 ± 78.2	3098.2 ± 39.5	0.285
Potassium (mg/d)	3019 ± 32.3	3491.8 ± 106.3	2968.7 ± 29.7	<0.001	91 ± 4.6	88 ± 3.6	0.001
Selenium (µg/d)	83.7 ± 1.1	91.6 ± 3.3	82.9 ± 1	0.009	1069 ± 92.9	1113.3 ± 61	0.303
Vitamin A (RETeq/d)	1119.5 ± 26.5	1177.3 ± 86	1113.4 ± 27.7	0.480	1.3 ± 0	1.2 ± 0	0.606
Vitamin B1 (mg/d)	1.2 ± 0	1.4 ± 0	1.2 ± 0	<0.001	1.7 ± 0.1	1.6 ± 0.1	0.029
Vitamin B2 (mg/d)	1.6 ± 0	1.8 ± 0.1	1.6 ± 0	0.005	18.8 ± 0.8	18.6 ± 0.3	0.406
Vitamin B3 (mg/d)	19.5 ± 0.3	21.5 ± 1	19.3 ± 0.3	0.027	5.1 ± 0.6	5.7 ± 0.4	0.758
Vitamin B5 (mg/d)	5 ± 0.1	5.7 ± 0.2	4.9 ± 0.1	0.003	5.2 ± 0.2	4.9 ± 0.1	0.244
Vitamin B6 (mg/d)	1.7 ± 0	2 ± 0.1	1.7 ± 0	<0.001	1.9 ± 0.1	1.7 ± 0	0.031
Vitamin B9 (µg/d)	281.8 ± 3.4	331.9 ± 11.1	276.5 ± 3.2	<0.001	331.3 ± 10.8	297.7 ± 6.4	0.001
Vitamin B12 (µg/d)	6 ± 0.2	6 ± 0.5	6 ± 0.2	0.967	2.5 ± 0.2	2.6 ± 0.2	0.220
Vitamin D (µg/d)	2.6 ± 0.1	2.6 ± 0.1	2.6 ± 0.1	0.875	11 ± 0.4	10.6 ± 0.3	0.480
Vitamin E (mg/d)	10 ± 0.2	11.3 ± 0.4	9.9 ± 0.2	<0.001	1.5 ± 0.1	1.3 ± 0	0.242

MUFA, Poly unsaturated fatty acids ; PUFA, Poly unsaturated fatty acids ; DHA, DocosaHexaenoic Acid ; EPA, Eicosapentaenoic acid

[1] *pvalue of the generalized linear model to test the difference between InAdeq and Adeq diets, accounting for the survey design without adjustment ;* [2] *pvalue of the generalized linear model to test the difference between InAdeq and Adeq diets, accounting for the survey design consumers vs non-consumers with adjustment on total energy intake, gender and the diet status*

Supplementary material

Table S5. Average total energy content, total quantity, nutritional indicators, environmental impact and estimated diet cost in observed diets not fulfilling the recommended guideline for pulses (InAdeq) and in substituted diets obtained with the STARCHES scenario (iso-portion replacement of starches by pulses until the recommended guideline for pulses is reached, i.e. 57g/d) and with the MEAT scenario (iso-portion replacement of meat by pulses until the recommended guideline for pulses is reached).

	**InAdeqObserved diets** (n = 1853)	**Substituted diets STARCHES** (n = 1853)		**Substituted diets MEAT** (n = 1853)		
	Mean ± *SE*	*Mean* ± *SE*	**Pvalue[1]**	*Mean* ± *SE*	**Pvalue[2]**	**Pvalue[3]**
Energy (kcal/d)	2058.2 ± 20.1	2033.8 ± 20.1	<.0001	2063.2 ± 20	<.0001	<.0001
Total quantity (g/d)	2632.2 ± 30.9	2632.2 ± 30.9	.	2663 ± 30.9	<.0001	<.0001
MAR (% adequacy)	81.2 ± 0.4	81.8 ± 0.4	<.0001	81.8 ± 0.4	<.0001	0.5315
MER (% excess)	136.6 ± 1.3	137 ± 1.3	<.0001	136 ± 1.3	<.0001	<.0001
SED (kcal/100g)	168.5 ± 1.3	166.2 ± 1.2	<.0001	164.2 ± 1.2	<.0001	<.0001
sPNNS-GS2	-0.6 ± 0.1	-0.1 ± 0.1	<.0001	0.1 ± 0.1	<.0001	<.0001
Proteins (% energy)	16.8 ± 0.1	17.3 ± 0.1	<.0001	16.4 ± 0.1	<.0001	<.0001
Carbohydrates (% energy)	44.3 ± 0.2	43.4 ± 0.2	<.0001	45.4 ± 0.2	<.0001	<.0001
Total fats (% energy)	36.5 ± 0.2	36.7 ± 0.2	<.0001	35.4 ± 0.2	<.0001	<.0001
Saturated fats (% energy)	14.6 ± 0.1	14.7 ± 0.1	<.0001	14.1 ± 0.1	<.0001	<.0001
Free sugars (% energy)	11.2 ± 0.2	11.3 ± 0.2	<.0001	11.1 ± 0.2	<.0001	<.0001
Sodium (g/d)	3567.5 ± 38.6	3663.3 ± 39.1	<.0001	3650.9 ± 38.9	<.0001	<.0001
Fibers (g/d)	18 ± 0.2	20.2 ± 0.2	<.0001	21.1 ± 0.2	<.0001	<.0001
Greenhouse gas emission (g CO2eq/d)	3891.3 ± 49.9	3934.7 ± 50.3	<.0001	3744.7 ± 47.1	<.0001	<.0001
Atmospheric acidification (g SO2eq/d)	48.5 ± 0.8	48.3 ± 0.8	<.0001	43.5 ± 0.7	<.0001	<.0001
Marine eutrophication (g Neq/d)	16.9 ± 0.2	17.6 ± 0.2	<.0001	16.9 ± 0.2	<.0001	<.0001
Estimated diet cost (€/d)	6.4 ± 0.1	6.4 ± 0.1	<.0001	6.2 ± 0.1	<.0001	<.0001

aa req, amino acids requirements; MAR, Mean adequacy ratio; MER, Mean excess ratio; SED, Solid energy density.

[1] pvalue of the generalized linear model to test the difference between STARCHES and observed diets, accounting for the survey design ; [2] pvalue of the generalized linear model to test the difference between MEAT and observed diets, accounting for the survey design ; [3] pvalue of the generalized linear model to test the difference between diets obtained with the STARCHES and MEAT scenarios, accounting for the survey design.

Supplementary material

Table S6. Bioavailability estimates and nutrient and bioavailability modulators contents in observed diets not fulfilling the recommended guideline for pulses (InAdeq) and in substituted diets obtained with the STARCHES scenario (iso-portion replacement of starches by pulses until the recommended guideline for pulses is reached, i.e. 57g/d) and with the MEAT scenario (iso-portion replacement of meat by pulses until the recommended guideline for pulses is reached.

	**Observed diets InAdeq** (n = 1853)	**Substituted diets STARCHES** (n = 1853)		**Substituted diets MEAT** (n = 1853)		
	Mean ± SE	*Mean ± SE*	**Pvalue[1]**	*Mean ± SE*	**Pvalue[2]**	**Pvalue[3]**
Iron (mg/d)	12.2 ± 0.1	12.9 ± 0.1	<.0001	12.6 ± 0.1	<.0001	<.0001
Heme iron (g/d)	1.5 ± 0	1.5 ± 0	<.0001	1.3 ± 0	<.0001	<.0001
Non-heme iron (g/d)	10.7 ± 0.1	11.3 ± 0.1	<.0001	11.4 ± 0.1	<.0001	<.0001
Positive modulators	±	±		±		
Total meat plus fish (g/d)	169.7 ± 3.1	169.1 ± 3.1	<.0001	149.8 ± 3	<.0001	<.0001
Vitamin C(g/d)	93.4 ± 1.7	92 ± 1.7	<.0001	93.2 ± 1.7	<.0001	<.0001
Negative modulators	±	±		±		
Phytate (mg/d)	932.7 ± 10.9	1023.5 ± 10.9	<.0001	1084.7 ± 11	<.0001	<.0001
Polyphenols from beverages (eq. cups of tea/d)	1.5 ± 0	1.5 ± 0	.	1.5 ± 0	.	.
Calcium (mg/d)	919.3 ± 10.9	928.4 ± 11	<.0001	935.3 ± 10.9	<.0001	<.0001
Heme absorption rate (%)	30.8 ± 0.2	30.8 ± 0.2	.	30.8 ± 0.2	.	.
Non-heme absorption rate (%)	4.5 ± 0.1	4.3 ± 0.1	<.0001	4.3 ± 0.1	<.0001	<.0001
Iron absorption rate (%)	7.6 ± 0.1	7.3 ± 0.1	<.0001	6.7 ± 0.1	<.0001	<.0001
Iron available for absorption (mg/d)	0.9 ± 0	0.9 ± 0	<.0001	0.8 ± 0	<.0001	<.0001
Zinc (mg/d)	10.4 ± 0.1	10.5 ± 0.1	<.0001	9.9 ± 0.1	<.0001	<.0001
Zinc absorption rate (%)	29 ± 0.2	28.1 ± 0.1	<.0001	28 ± 0.1	<.0001	<.0001
Zinc available for absorption (mg/d)	2.9 ± 0	2.8 ± 0	<.0001	2.6 ± 0	<.0001	<.0001
Histidine (% requirement)	170.6 ± 0.3	171.9 ± 0.3	<.0001	169.3 ± 0.3	<.0001	<.0001
Isoleucin (% requirement)	161.9 ± 0.2	162.2 ± 0.2	<.0001	161.7 ± 0.2	<.0001	<.0001
Leucine (% requirement)	128.8 ± 0.1	128.9 ± 0.1	<.0001	128.7 ± 0.1	<.0001	<.0001
Lysin (% requirement)	146.7 ± 0.4	148.4 ± 0.4	<.0001	144.9 ± 0.4	<.0001	<.0001
Sulfur amino acids (% requirement)	156.9 ± 0.2	154.7 ± 0.2	<.0001	154.5 ± 0.2	<.0001	<.0001
Aromatic amino acid (% requirement)	187.2 ± 0.2	187.1 ± 0.2	<.0001	188.3 ± 0.2	<.0001	<.0001
Threonin (% requirement)	152.4 ± 0.2	153 ± 0.2	<.0001	151.3 ± 0.2	<.0001	<.0001
Tryptophan (% requirement)	191.4 ± 0.2	190.2 ± 0.2	<.0001	191.3 ± 0.2	<.0001	<.0001
Valin (% requirement)	188.1 ± 0.2	188 ± 0.2	0.0804	188.5 ± 0.2	<.0001	<.0001
PDCAAS	94.9 ± 0.02	94.5 ± 0.02	<.0001	94.5 ± 0.03	<.0001	0.7901

aa req, amino acids requirements; PDCAAS, Protein Digestibility Corrected Amino Acid Score.

[1] pvalue of the generalized linear model to test the difference between STARCHES and observed diets, accounting for the survey design ; [2] pvalue of the generalized linear model to test the difference between MEAT and observed diets, accounting for the survey design ; [3] pvalue of the generalized linear model to test the difference between diets obtained with the STARCHES and MEAT scenarios, accounting for the survey design.
